# Pediatric Gastrointestinal Endoscopy: Diagnostic Yield and Appropriateness of Referral Based on Clinical Presentation: A Pilot Study

**DOI:** 10.3389/fped.2021.607418

**Published:** 2021-10-29

**Authors:** Tahel Fachler, Eyal Shteyer, Esther Orlanski Meyer, Ibrahim Shemasna, Raffi Lev Tzion, Yelena Rachman, Ari Bergwerk, Dan Turner, Oren Ledder

**Affiliations:** ^1^Shaare Zedek Medical Center, Jerusalem, Israel; ^2^The Hebrew University of Jerusalem, Jerusalem, Israel

**Keywords:** indications, resource allocation, diagnostic yield, pediatric, gastrointestinal endoscopy

## Abstract

**Objectives:** There is a lack of evidence-based consensus for the utility of gastrointestinal endoscopy (GIE) in an array of frequently occurring symptoms in children. We aimed to assess the diagnostic yield of endoscopy in an effort to aid clinical decision making.

**Methods:** Retrospective analysis included patients ≤18 years who underwent GIE during one calendar year at Shaare Zedek Medical Center. We excluded children referred for predefined obvious indications for GIE, planned follow-up procedures, and therapeutic endoscopy. Clinician-assigned indication for endoscopy as well as endoscopic and histologic findings were recorded. Diagnostic yield of GIE was determined according to referral indication.

**Results:** There were 794 endoscopies performed of which 329 were included in the analysis (mean age 9.3 ± 5.0 years, 51% female). No significant complications of GIE were recorded. Six major referral indications were identified among which abdominal pain was the most frequent 88/329 (26%) of whom 32/88 (36%) had a significant diagnostic finding. Among the other major indications, diagnostic findings were found in 36/85 (43%) children with primary indication of chronic diarrhea, 14/33 (42%) failure to thrive, 15/32 (46%) short stature, 30/56 (54%) iron deficiency, and 20/48 (42%) weight loss.

**Conclusions:** Pediatric GIE is a safe procedure with diverse clinical indications. The diagnostic yield of endoscopy is variable, depending on the referral indication. These data can assist formulating judicious referral practices.

## Highlights

- Gastrointestinal endoscopy (GIE) is a safe and useful diagnostic intervention in children.
- There exist multiple clinical scenarios for which its utility remains uncertain.
- The majority of recommendations in the ESPGHAN/ESGE guidelines of pediatric GIE are weak with low quality of evidence.
- Diagnostic yield ranged significantly based on the referral indication.
- Low yield for abdominal pain (AP) with diarrhea and AP with constipation if blood results are normal.
- High yield for AP with iron deficiency, chronic diarrhea with weight loss, and isolated iron deficiency.
- There is a higher diagnostic yield with objective indications than subjective symptoms.

## Introduction

Gastrointestinal endoscopy (GIE), including esophagogastroduodenoscopy (EGD) and colonoscopy, has become an integral component of diagnosis and therapeutics in pediatric gastroenterology. Although there is some overlap in referral indications between adults and children, significant differences exist. Screening colonoscopies, for example, provide the critical mass of endoscopic investigations in adults, whereas gastroscopy for suspected celiac is proportionally more frequent in children (1).

Unlike in adults (2), until recently, there was a dearth of evidence-based guidelines of appropriate indications for endoscopic evaluations in children. Although there are clinical scenarios that are considered an absolute indication for GIE, such as significant upper gastrointestinal bleeding, the correct placement of GIE for a multitude of clinical scenarios has not been formalized. As a consequence of the lack of guidelines, endoscopies may be performed inappropriately with resultant patient inconvenience and cost burden (3).

In this study, we aimed to assess the diagnostic yield of pediatric GIE in various clinical scenarios based on symptoms, signs, and laboratory findings. Determination of the diagnostic yield of endoscopy by indication of referral could facilitate more judicious decision making as to which patients would benefit from an endoscopic procedure.

## Methods

### Patient Population

We conducted a retrospective review of all pediatric GIEs performed during calendar year 2015 at Shaare Zedek Medical Center in Jerusalem. Recorded data included demographics, referral source, and clinical features, including presenting symptoms and anthropomorphic data, laboratory results, endoscopic findings, and histology. The indication for referral to GIE was determined from either the referral letter completed by the referring pediatric gastroenterologist and/or from the previous clinic visit summary.

Under our aim of determining the diagnostic yield of endoscopy in clinical scenarios of uncertainty, we excluded endoscopies that were undertaken for what we defined as obvious indications, including significantly elevated celiac serology [≥3x upper limit of normal (ULN)], significant UGI bleed, and lower GI bleed in the absence of clinical suspicion of constipation. Similarly, scheduled follow-up procedures and therapeutic endoscopies, such as foreign body impaction, stricture dilatations, or esophageal varices, were excluded from analysis. This study was reviewed and approved by the Shaare Zedek Medical Center Helsinki ethics committee.

### Endoscopic Procedures

The endoscopic procedures were performed as per routine protocol under general anesthesia. *Helicobacter pylori* was assessed by hematoxylin & eosin and Giemsa staining.

Endoscopic or histologic findings were considered significant if they had diagnostic or prognostic value, defined as a reasonable explanation for presenting symptoms, and/or a finding that effects management change. Minor, non-specific endoscopic findings, such as subtle erythema, minor increase or decrease of vascularity, or mild pallor were considered normal if there were no corresponding histologic changes of significance. Similarly, minor, non-specific histologic findings, such as mild chronic gastritis with no activity, were considered normal if seemingly unrelated to the presenting indication (4–6). Borderline results were defined as a histological abnormality of questionable significance, for example, mild, non-specific duodenitis or mild basal hyperplasia of esophageal mucosa with no associated inflammation. Incidental findings unrelated to the referral indication, for example, *H. pylori* in a patient referred with diarrhea, were noted but not considered a positive find.

## Results

**Figure 1:**
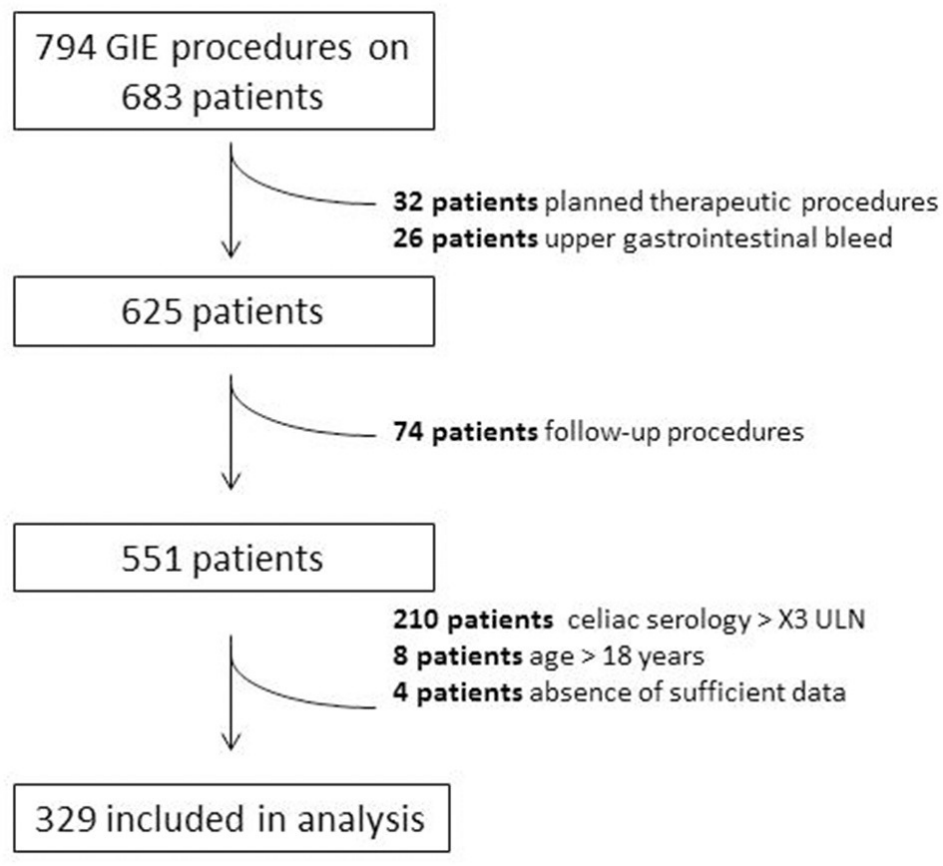
Selection of patients included in study.

**Figure 2:**
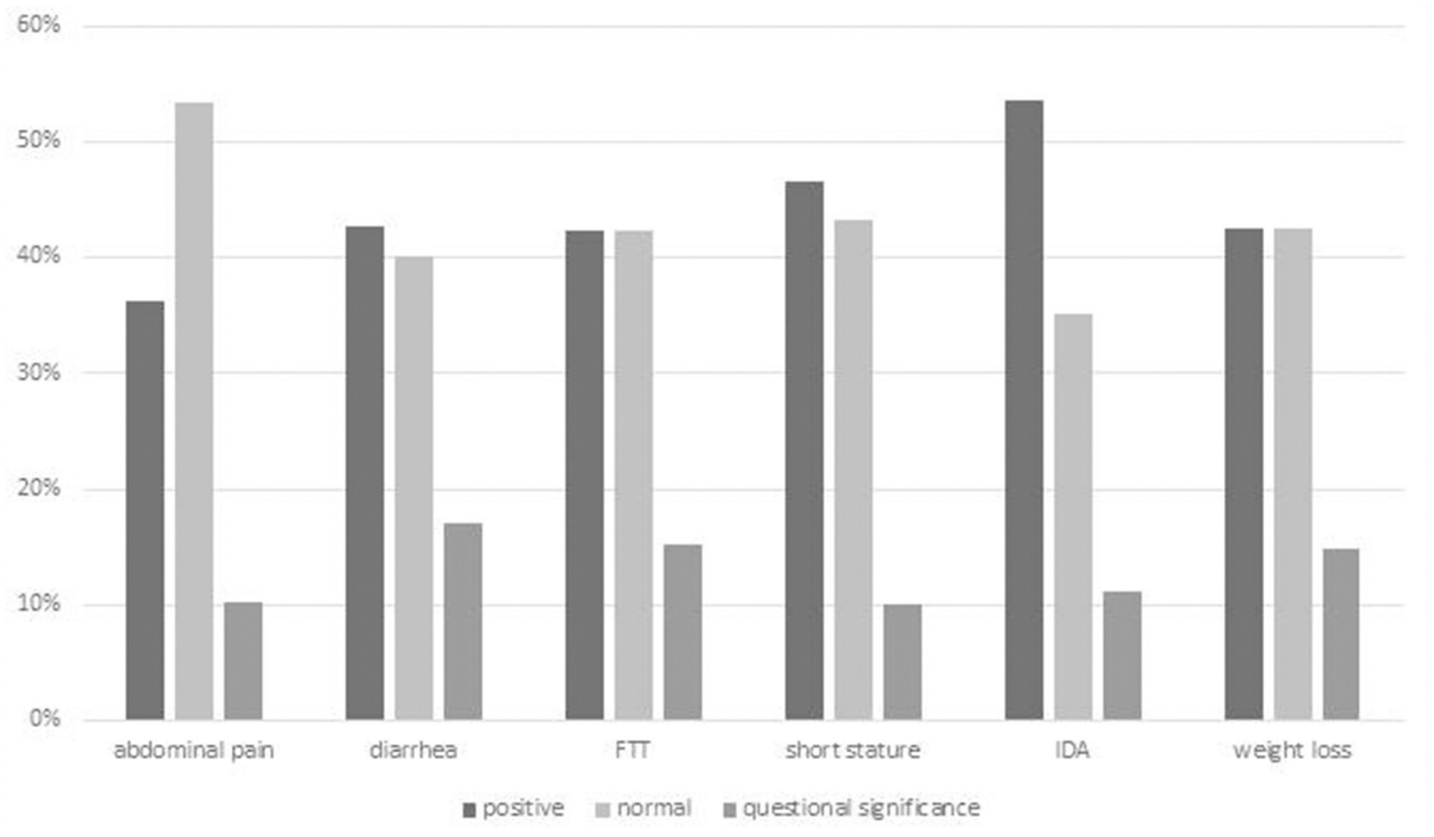
Overall diagnostic yield of GIE by indication. Positive finding = definite diagnostic finding relating to indication; Questionable significance = finding of uncertain relationship to indication or minor finding of uncertain significance. FTT, failure to thrive.

A total of 794 GIE procedures were performed on 683 individuals among which 329 met criteria for inclusion (Figure 1). Mean age of included children was 9.3 ± 5.0 years (range 0–18), 51% female. Of the 329 procedures, 273 (78%) underwent EGD only, five (3%) underwent colonoscopy only, and 51 (19%) underwent both EGD and colonoscopy. No major procedure-related complications of GIE, such as postendoscopy bleeding, bowel perforation, or unplanned postendoscopy admissions, were recorded. We identified six major indications for GIE: abdominal pain, diarrhea, failure to thrive (FTT), short stature, iron deficiency, and weight loss. Patients with referral indications other than these are described separately. In the majority of these indications, the diagnostic yield was above 40% (Supplementary Table 1; Figure 2).

### Abdominal Pain

**Figure 3:**
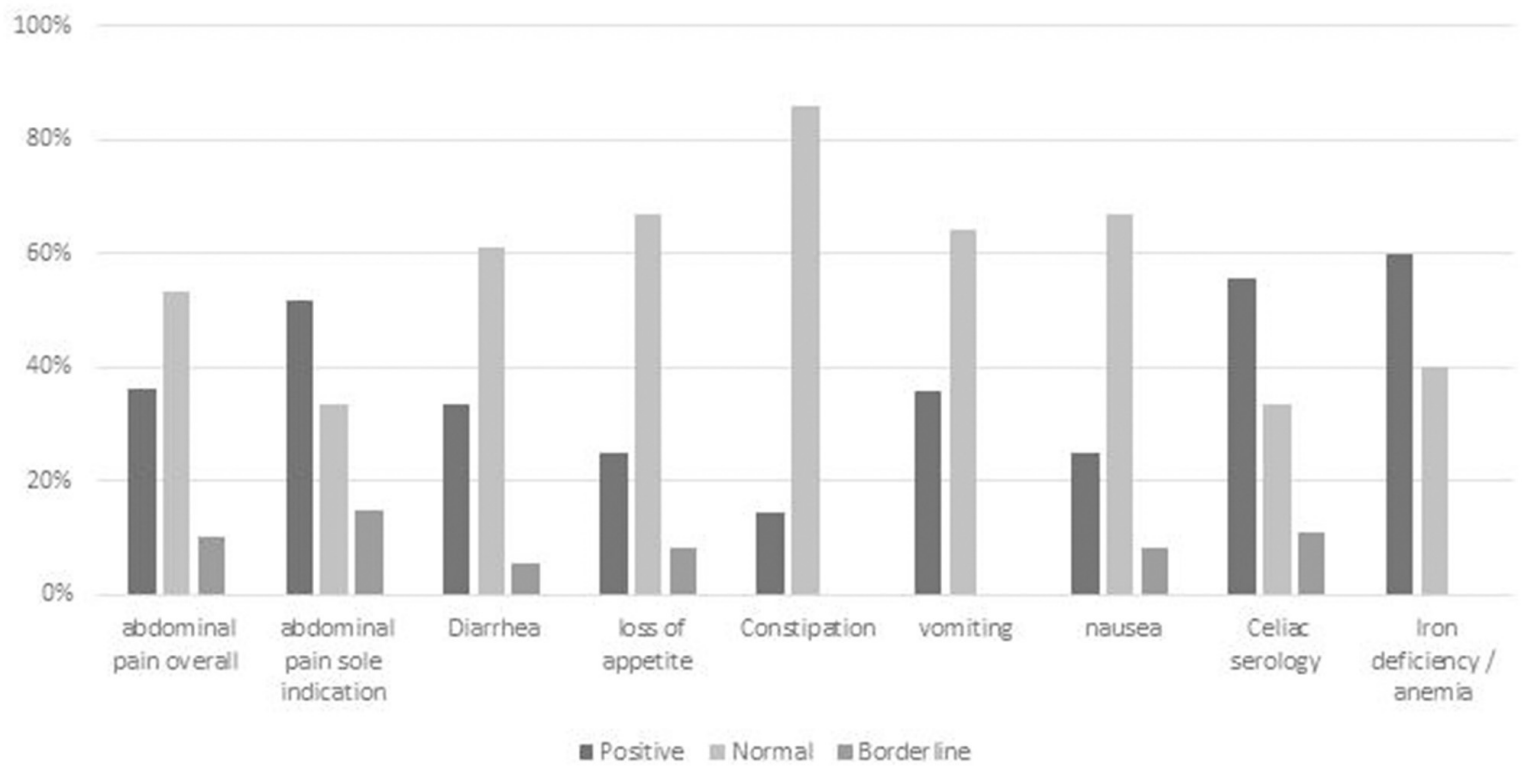
Diagnostic yield of GIE in subgroup analysis of abdominal pain with different joint referral indications. Positive = definite diagnostic finding relating to indication; Borderline = finding of uncertain relationship to indication or minor finding of uncertain significance; Negative = no finding or incidental finding unrelated to indication. AP, abdominal pain; celiac serology < x3 ULN.

Eighty-eight children (26% of the cohort) underwent GIE with abdominal pain as a major indication (Figure 3). Overall, 32/88 (36%) children had a diagnostic finding relating to abdominal pain. Incomplete descriptions in patient notes precluded the ability to analyze epigastric pain independently. Children in whom abdominal pain was a sole indication had a similar rate of findings as those with joint indications. Among those joint indications in which the positive diagnostic yield was <25% were constipation, loss of appetite, and nausea. Patients with abdominal pain and iron deficiency and/or weakly positive celiac serology had diagnostic findings in more than 50% (Supplementary Table 1).

### Chronic Diarrhea

**Figure 4:**
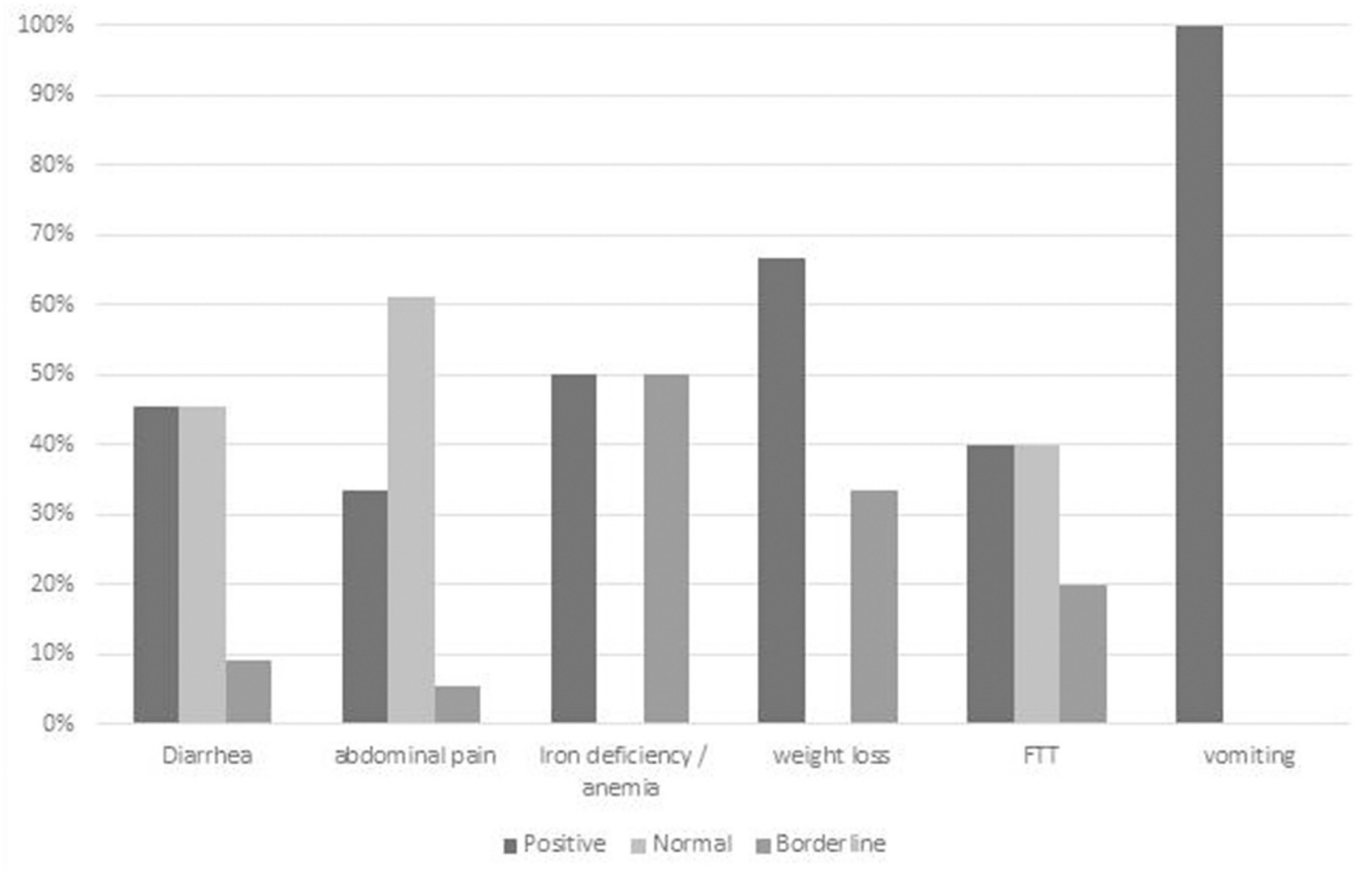
Diagnostic yield of GIE in subgroup analysis of chronic diarrhea with different joint referral indications. Positive = definite diagnostic finding relating to indication; Borderline = finding of uncertain relationship to indication or minor finding of uncertain significance; Negative = no finding or incidental finding unrelated to indication. IDA, iron deficiency anemia; FTT, failure to thrive.

Eighty-five children (26% of the cohort) presented with chronic diarrhea as a major indication. Overall 36 (43%) had a diagnostic finding relating to chronic diarrhea. Eleven children (3%) underwent GIE in which chronic diarrhea was the sole indication (Supplementary Table 1; Figure 4), five (45%) of whom had diagnostic biopsies related to diarrhea. Children with chronic diarrhea and iron deficiency anemia had a positive finding identified in 3/6 (50%). Diagnostic findings related to diarrhea were obtained in 2/5 (40%) children who presented with chronic diarrhea and FTT.

### Iron Deficiency Anemia

Fifty-six children (17% of the cohort) underwent endoscopy with iron deficiency as a major indication, of whom 30 (54%) had a positive diagnostic finding related to the indication. Among those patients referred with iron deficiency anemia and weakly positive celiac serology, celiac disease was confirmed in 4/6 (67%) (Supplementary Table 1).

### Failure to Thrive

Thirty-three endoscopies (10% of the cohort) were performed with an indication of FTT, in eight (25%) of whom FTT was the only indication. Overall, positive diagnostic findings relating to FTT were identified in 14 (42%) of these patients. FTT with a joint indication of iron deficiency anemia had diagnostic findings in 6/12 (50%) (Supplementary Table 1).

### Short Stature

Thirty-two children (10% of the cohort) underwent endoscopy for a primary indication of short stature. Positive findings were recorded for nine (29%) of these patients, all of whom had a second indication besides short stature. Among the 11 children who presented with short stature and weakly positive celiac serology, five (45%) were diagnosed with celiac based on histology (Supplementary Table 1).

### Weight Loss

Forty-eight children (15% of the cohort) were referred due to unexplained weight loss as a major indication, among whom 20 (42%) had diagnostic findings. Six (19%) of these children were diagnosed with IBD, all of whom had a secondary indication besides weight loss. In those children with both weight loss and iron deficiency anemia, the diagnostic yield increased to 5/9 (56%) (Supplementary Table 1).

### Weakly Positive Celiac Serology

Celiac serology ≥3x ULN was excluded from analysis as per the study protocol. Among the 329 endoscopies included in the analysis, 35 (11%) patients had borderline celiac serology of <3x ULN. Of these, two (6%) children had no other indication with the others presenting with joint indications of abdominal pain, short stature, iron deficiency, diarrhea, weight loss, and/or FTT. Two (6%) of these children had IgA deficiency with borderline IgG-based serology.

Twenty-two (63%) of these children had histologic features consistent with celiac, and four (11%) had borderline histology. Celiac was more likely in children with additional indications, such as iron deficiency (67%) and abdominal pain (56%), than in children with FTT (21%) or weight loss (4%) (Supplementary Table 1).

### Miscellaneous

Other than the major indications described previously, there were a few other indications that had a very high rate of positive findings. Family history of celiac disease, combined with weakly positive celiac serology, yielded positive histology in eight out of nine (89%) children. Similarly, there were five children referred due to fatigue and weakly positive celiac serology among whom celiac was diagnosed in three (60%) of these children.

All children diagnosed with celiac disease over 3 years of age had either borderline or weakly elevated serology. All patients diagnosed with IBD had at least one abnormal blood result, such as anemia, hypoalbuminemia, and/or raised inflammatory markers.

Overall, the diagnostic yield of GIE ranged significantly based on the referral indication, from 14% (abdominal pain with constipation) to 67% (chronic diarrhea with weight loss). Various presentations or combinations of symptoms had a particularly low yield of positive findings, including abdominal pain with constipation 1/7 (14%), abdominal pain with diarrhea 6/18 (33%), and short stature 2/8 (25%) in the absence of clinical or biochemical suggestion of celiac or IBD.

The difference between the diagnostic yield of subjective symptoms such as loss of appetite 6/36 (17%), constipation 9/29 (31%), and nausea 3/12 (25%) was consistently lower than the diagnostic yield of more objective clinical indications, such as iron deficiency anemia 30/56 (54%) and slightly increased celiac serology 22/35 (63%).

## Discussion

The utility of GIE has expanded tremendously in pediatric gastroenterology, including a 1,200% increase at one large center over the 20-year period till 2005 (7) and a 400% increase over the last decade at the authors' center (unpublished data). With the associated burgeoning costs, it is important to utilize this service efficiently and minimize unnecessary investigations in those children with a low pretest probability of finding any significant pathology.

In our study, diagnostic yield ranged significantly based on the referral indication with a low diagnostic yield for joint indications of abdominal pain and diarrhea, constipation, loss of appetite, and nausea in the absence of significant abnormal blood results and a high diagnostic yield in children with abdominal pain and iron deficiency, chronic diarrhea with weight loss or vomiting, and also in isolated iron deficiency.

The indications for GIE have changed over time, being initially reserved for more critical circumstances. GI bleeding made up 34% of all procedures in 1985 compared with only 5% in 2005 with an increase in procedures performed for abdominal pain over the same time period from 23 to 43% (7).

Despite its widespread use, there remains a lack of consensus as to the appropriate indications for GIE in children. The overwhelming majority of the recommendations in the European Society for Pediatric Gastroenterology Hepatology and Nutrition (ESPGHAN) and the European Society of Gastrointestinal Endoscopy (ESGE) guidelines of pediatric GIE are weak with low quality of evidence (8).

To address this need, we analyzed all GIE procedures from a relatively large-volume pediatric service to analyze the diagnostic yield of GIE, specifically in those cases in which consensus is lacking. With the aim of identifying diagnostic yield in those circumstances of greater doubt, we excluded planned follow-up procedures, therapeutic procedures, and procedures predefined as “necessary” by consensus.

Diagnostic yield of GIE in relation to presenting symptoms has been reviewed in several previous studies (3, 9–13); however, there is significant variability between these data. Some studies analyze all GIE procedures, and others only diagnostic procedures, and another only those performed for abdominal pain. Furthermore, the definition of a positive finding was not uniform with some studies including all findings, others histologic findings, and another including only those findings that led to a change of diagnosis and/or management. The resultant diagnostic yield ranged from 19 to 76% overall. When specifically assessing those cases referred for abdominal pain, the diagnostic yield ranged from 38 to 69%. In comparison, our study tended to a lower diagnostic yield than in most previous studies, which was not surprising considering our targeted analysis excluded those patients in whom consensus would suggest the need for the procedure.

Some findings are of uncertain significance to the referral indication, and others are clearly incidental. An example of this is *H. pylori*, in which, with carriage rates upward of 40% in young adults in Israel (14), this is frequently an incidental finding. There is uncertainty about the relationship between *H. pylori* and both abdominal pain and iron deficiency in the absence of significant endoscopic gastritis or ulcerations (15–21). Despite conflicting data, we assumed *H. pylori* to be clinically significant in our study, when the procedure was performed for abdominal pain or iron deficiency.

This study's main limitation is its retrospective nature in which the need to represent complex combinations of patient findings into simple, defined referral indications remains a challenge. As such, due to multiple permutations and combinations of clinical features, despite the large number of children included in our study, only small numbers were represented in some clinical scenarios. Additionally, the number of sole colonoscopies was relatively low, and this seemingly reflects pediatric endoscopy practice. These limitations may have been overcome somewhat with a larger sample size; however, expanding the number of included procedures beyond what was included was not possible in this study. As such, this publication should be seen to pave the way for larger and more powerful studies, preferably of a prospective nature, to further address the study question. Furthermore, laboratory data was not universally available for all patients, precluding comprehensive statistical analysis and limiting conclusions to more general qualitative outcomes as described. Regardless, our study is one of the largest studies to address this question and the largest to include only those patients in whom GIE would not be considered an absolute requirement.

This study makes an important contribution in identifying indications for pediatric endoscopy that have a relatively higher diagnostic yield and can assist the clinician in deciding on the need for diagnostic GIE in these common scenarios. The ultimate responsibility for deciding on recommending GIE to a patient rests with the clinician based on his or her experienced assessment of the patient's symptoms, signs, and laboratory results. Our data, combined with previously published data, assists the clinician to refer patients more judiciously.

## Data Availability Statement

The raw data supporting the conclusions of this article will be made available by the authors, without undue reservation.

## Author Contributions

TF: acquisition, analysis and interpretation of data, and drafting manuscript. ES, EO, IS, RL, YR, AB, and DT: data acquisition and critical review of manuscript. OL: conception and design of the study, analysis and interpretation of data, drafting manuscript, and final approval of version to be published. All authors contributed to the article and approved the submitted version.

## Conflict of Interest

The authors declare that the research was conducted in the absence of any commercial or financial relationships that could be construed as a potential conflict of interest.

## Publisher's Note

All claims expressed in this article are solely those of the authors and do not necessarily represent those of their affiliated organizations, or those of the publisher, the editors and the reviewers. Any product that may be evaluated in this article, or claim that may be made by its manufacturer, is not guaranteed or endorsed by the publisher.
