# Supplementary material for: Pediatric Gastrointestinal Endoscopy: Diagnostic Yield and Appropriateness of Referral Based on Clinical Presentation: A Pilot Study
Source: Front Pediatr. 2021 Oct 29;9:607418. doi: 10.3389/fped.2021.607418 (PMC8586696; doi:10.3389/fped.2021.607418)
Supplement: Supplementary file 1 [file Table_1.DOCX]

**Supplemental Table 1:** Diagnostic findings relating to indication

| **Indication** | **Sub-indication** | **Diagnosis** | **Frequency (%)** |
| --- | --- | --- | --- |
| **Abdominal pain (n=88)** | AP as sole indication (n=27) | H. pylori | 11 (40.7%) |
|  |  | EoE | 3 (11.1%) |
|  |  | GERD | 1 (3.7%) |
|  |  | No finding | 9 (33.3%) |
|  | AP and diarrhea (n=18) | H. pylori | 3 (16.7%) |
|  |  | GERD | 3 (16.7%) |
|  | AP and constipation (n=7) | H. pylori | 1 (14.3%) |
|  |  | normal | 6 (85.7%) |
|  | AP and vomiting (n=14) | H. pylori | 3 (21.4%) |
|  |  | GERD | 1 (7.1%) |
|  |  | Duodenitis | 1 (7.1%) |
|  |  | No finding | 9 (64.3%) |
|  | AP and nausea (n=12) | H. pylori | 2 (16.7%) |
|  |  | GERD | 1 (8.3%) |
|  |  | Mild duodenitis (borderline) | 1 (8.3%) |
|  |  | No finding | 8 (66.7%) |
|  | AP and weakly positive celiac serology (n=9) | Celiac | 5 (55.6%) |
|  |  | Mild duodenitis | 1 (11.1%) |
|  |  | No finding | 3 (33.4%) |
|  | AP and iron deficiency anemia (n=5) | H. pylori | 1 (20%) |
|  |  | GERD | 1 (20%) |
|  |  | IBD | 1 (20%) |
|  |  | No finding | 2 (40%) |
|  | AP and loss of appetite (n=12) | H. pylori | 2 (16.7%) |
|  |  | IBD | 1 (8.4%) |
|  |  | No finding | 8(66.7%) |
|  |  | Mild duodenitis(borderline) | 1 (8.4%) |
|  | AP and FTT (n=1) | No finding | 1 (100%) |
|  |  |  |  |
| **Chronic diarrhea (n=97)** | Chronic diarrhea sole indication (n=11) | Celiac | 2 (18.2%) |
|  |  | IBD | 2 (18.2%) |
|  |  | Microvillus inclusion disease | 1 (9.1%) |
|  |  | No finding | 5 (45.5%) |
|  |  | Mild duodentitis (borderline) | 1 (9.1%) |
|  | Chronic diarrhea and IDA (n=6) | Celiac | 2 (33.4%) |
|  |  | IBD | 2 (33.4%) |
|  |  | H. pylori | 1 (16.7%) |
|  |  | mild duodenitis (borderline) | 3 (50%) |
|  | Chronic diarrhea and weight loss (n=3) | IBD | 1 (33.4%) |
|  |  | celiac | 1 (33.4%) |
|  |  | No finding | 1 (33.4%) |
|  | Chronic diarrhea and vomiting (n=1) | Celiac | 1 (100%) |
|  | Chronic diarrhea and FTT (n=10) | Celiac | 1 (10%) |
|  |  | No finding | 9 (90%) |
|  |  |  |  |
| **FTT (n=33)** | FTT as sole indication (n=6) | Celiac | 1 (16.7%) |
|  |  | EoE | 1 (16.7%) |
|  |  | GERD | 1 (16.7%) |
|  |  | H pylori | 1 (16.7%) |
|  |  | No finding | 2 (33.3%) |
|  | FTT and IDA (n=12) | Celiac | 5 (41.7%) |
|  |  | H. pylori | 4 (33.4%) |
|  |  | No finding | 3 (25%) |
|  | Mild duodenitis | borderline | 3 (25%) |
|  | FTT and abdominal pain (n=7) | Celiac | 2 (28.6%) |
|  |  | GERD | 1 (14.3%) |
|  |  | H. pylori | 1 (14.3%) |
|  |  | Mild duodenitis | 1 (14.3%) |
|  |  | No finding | 3 (42.9%) |
| **Short stature (n=32)** |  | Celiac | 7 (21.9%) |
|  |  | IBD | 2 (6.2%) |
|  |  | No finding | 23 (71.9%) |
|  |  |  |  |
| **Weight loss (n=48)** | Weight loss as sole indication (n=6) | EoE | 1 (16.7%) |
|  |  | No finding | 5 (83.3%) |
|  | Weight loss and diarrhea and/or AP (n=33) | IBD | 5 (15.2%) |
|  |  | Celiac | 1 (3%) |
|  |  | GERD | 2 (6.1%) |
|  |  | No finding | 25 (75.8%) |
|  |  |  |  |
| **IDA (n=56)** | IDA as sole indication (n=9) | Celiac | 1 (11.1%) |
|  |  | IBD | 1 (11.1%) |
|  |  | EoE | 1 (11.1%) |
|  |  | Collagenous gastritis | 1 (11.1%) |
|  |  | No finding | 5 (55.6%) |

AP – abdominal pain, GERD – gastroesophageal reflux disease, IBD – inflammatory bowel disease, IDA – iron deficiency anemia, FTT – failure to thrive, EoE – eosinophilic esophagitis.

No finding = no definite diagnostic finding relating to indication

Note: Some patients had several indications and some had more than one diagnostic finding. Patients with celiac serology >3x ULN were excluded from analysis as per study protocol
